# MdNup62 involved in salt and osmotic stress tolerance in apple

**DOI:** 10.1038/s41598-023-47024-9

**Published:** 2023-11-18

**Authors:** Ruxuan Guo, Xiaoshuang Zhang, Mingyuan Li, Huiwen Zhang, Junkai Wu, Libin Zhang, Xiao Xiao, Mingyu Han, Na An, Libo Xing, Chenguang Zhang

**Affiliations:** 1 Hebei Key Laboratory of Horticultural Germplasm Excavation and Innovative Utilization, Hebei Higher Institute Application Technology Research and Development Center of Horticultural Plant Biological Breeding, College of Horticulture Technology, Hebei Normal University of Science and Technology, Changli, 066600 Hebei People’s Republic of China; 2 College of Horticulture, Northwest A&F University, Yangling, 712100 Shaanxi People’s Republic of China

**Keywords:** Plant sciences, Environmental sciences

## Abstract

Abiotic stress of plants has serious consequences on the development of the apple industry. Nuclear pore complexes (NPCs) control nucleoplasmic transport and play an important role in the regulation of plant abiotic stress response. However, the effects of NPCs on apple salt and osmotic stress responses have not been reported yet. In this study, we analyzed the expression and function of *NUCLEOPORIN 62* (*MdNup62)*, a component of apple NPC. *MdNup62* expression was significantly increased by salt and mannitol (simulated osmotic stress) treatment. The *MdNup62*-overexpressing (OE) *Arabidopsis* and tomato lines exhibited significantly reduced salt stress tolerance, and *MdNup62*-OE *Arabidopsis* lines exhibited reduced osmotic stress tolerance. We further studied the function of HEAT SHOCK FACTOR A1d (MdHSFA1d), the interacting protein of MdNup62, in salt and osmotic stress tolerance. In contrast to *MdNup62*, *MdHSFA1d*-OE *Arabidopsis* lines showed significantly enhanced tolerance to salt and osmotic stress. Our findings suggest a possible interaction of MdNup62 with MdHSFA1d in the mediation of nuclear and cytoplasmic transport and the regulation of apple salt and osmotic stress tolerance. These results contribute to the understanding of the salt and osmotic stress response mechanism in apple.

## Introduction

Plant growth and development are limited by osmotic stress, which is caused by various factors, including high salinity and drought. Osmotic stress is defined as an imbalance of osmotic potential between plants and their environment resulting in injury to cells or plants. Soil salinization, as a cause of osmotic stress, is a global problem and affects approximately 8.31 × 10^8^ hm^2^ of soil resources^1^. Therefore, it is important to cultivate crop varieties with salt- and osmotic-stress tolerance.

The nucleus is the main locus of genetic and metabolic regulation in eukaryotes. The nuclear pore complex (NPC) is the main channel of communication between the nucleus and cytoplasm. The NPC is composed of nucleoporins, among which, 38 components have been identified in apple to date^2^. According to the position and function of nucleoporins in the nuclear pore, some nucleoporins are composed of three subcomplexes: Nup62, Nup93, and Nup107–160^2, 3^. These nucleoporins control the passage of RNA, protein, and other macromolecules into and out of the nucleus and maintain the normal life activities of cells, which, in turn, plays a crucial role in growth and development^4^. Nucleoporins regulate numerous plant life activities, such as flowering^5–8^, immunity^9–11^, hormones^12, 13^, and abiotic stress pathways^14, 15^. Under heat stress, the hypocotyl elongation, survival rates of seedlings and inflorescences, membrane integrity, and photosystem II activity (Fv/Fm) of the nucleoporin *PATHOGENGSIS-RELATED GENES 5* (*cpr5)* mutant were significantly reduced. However, after transforming *CPR5* gene into the *cpr5* mutant line, its heat tolerance was restored^16^. Nup85 and Nup133 only control mRNA output under warm conditions, and are more sensitive to the localization of transcription factors under warm conditions. In addition, Nup96 and HIGH EXPRESSION OF OSMOTICALLY RESPONSIVE GENES 1 (HOS1) play key roles in maintaining high expression levels of high-temperature response genes by promoting the nuclear accumulation of PHYTOCHROME INTERACTING FACTOR 4 (PIF4) at warm temperatures^4^. *Nup85* mutant can reduce the expression of stress response genes and increase the sensitivity to ABA- and salt-stress^15^.

Plant heat shock factors (HSFs) play an important role in signal transduction, regulation of plant growth and development, and various stress pathways^17^. Consistent with the nomenclature of HSF, the heat tolerance function was the first and most widely studied in plants. Several plant *HSFs* are involved in the regulation of high temperature tolerance, such as *HSFA1*, *HSFA2*, *HSFA9*, *HSFB1*, and *HSFB2*^18–21^. Additionally, *HSFs* are also involved in drought and salt stress responses. Under drought stress, the HEAT SHOCK PROTEIN 90 (MdHSP90)-MdHSFA8a complex dissociates to release MdHSFA8a, which further interacts with RELATED TO AP2 12 (RAP2.12) to activate downstream gene activity and enhance plant survival^22^. The drought- and salt-tolerance of *OsHSFB2b* overexpressing plants decreased significantly, whereas the tolerance of *OsHSFB2b*-RNAi lines increased significantly^23^. *Arabidopsis AtHSFA6a* is also involved in the regulation of salt-tolerance and drought-tolerance pathways^24^.

Apple (*Malus* × *domestica Borkh.*) is widely cultivated in temperate regions of the world and is an economically important fruit tree. However, due to global climate change, salt stress presents serious challenges to the development of the apple industry. Therefore, it is important to strengthen the research on related molecular mechanisms. To date, there have been few reports on the molecular mechanism of salt-tolerance in apple. Genes, such as *C-REPEAT BINDING FACTORS* (*MdCBF*), *AUTOPHAGY-RELATED 10* (*MdATG10*), *Na*^+^*-H*^+^ *EXCHANGER* 1 (*MdNHX1*), and *ETHYLENE RESPONSE FACTORS* 3/4 (*MdERF3/4*) are involved in the regulation of salt stress response in apple^25–28^. However, the molecular regulatory network in response to salt stress is relatively complex, and the research on apple is underdeveloped and difficult to conduct. Further research on salt tolerance of apple is required.

Our previous studies show apple MdNup62 interacts with several MdHSFAs and participates in the heat stress response pathway^29^. In the present study, we found that apple MdNup62 and MdHSFA1d were significantly induced by salt and mannitol treatment. And the salt and osmotic stress tolerance of *MdNup62*-OE plants were significantly reduced, whereas the *MdHSFA1d*-OE plants were significantly increased. These findings suggest that both *MdNup62* and *MdHSFA1d* are involved in the regulation of salt and osmotic stress responses in apple.

## Materials and methods

### Plant materials and growth conditions

‘Nagafu No. 2′ plants were grown on MS medium containing 0.1 mg L^−1^ indolebutyric acid and 0.6 mg L^−1^ 6-benzylaminopurine under long-day conditions (16 h-light/8 h-dark) at 24 °C and were sub-cultured every 45 d. *Arabidopsis* plants (‘Columbia’) were grown on 1/2MS medium under long-day conditions (16 h-light/8 h-dark) at 22 °C. And the components of 1/2MS plates are MS 2.2 g/L, Agar 7 g/L, Sucrose 30 g/L. Tomato plants (‘Ailsa Craig’) were grown on MS medium under long-day conditions (16 h-light/8 h-dark) at 25 °C. And the components of MS plates are MS 4.4 g/L, Agar 7 g/L, Sucrose 30 g/L. *Arabidopsis* and tomato seeds were previously preserved in our laboratory.

### Protein alignment and phylogenetic relationship analysis

We downloaded HSFA1d protein sequences of nine plant species (*Arabidopsis*, *Malus domestica*, *Populus trichocarpa*, *Oryza sativa*, *Rosa chinensis*, *Pyrus bretschneideri*, *Ananas comosus*, *Prunus dulcis*, and *Glycine max*) from NCBI. And a protein sequence alignment of these nine plant species was performed using DNAMAN software. A phylogenetic tree comprising HSFA1d from nine species was constructed using the MEGA-X program.

### RNA extraction and qRT-PCR analysis

Total RNA was extracted from apple and *Arabidopsis* seedlings using an RNA Plant Plus Reagent Kit (TIANGEN, Beijing, China). cDna was synthesized from the RNA using a PrimeScript RT Reagent Kit (Takara, Shiga, Japan). qRT-PCR analysis was conducted on a StepOnePlus Real-Time PCR System (Thermo Fisher Scientific, USA). The reaction solution contained 10 μL of SYBR Green I Master Mix (CWBIO, Beijing, China), 0.5 μmol L^−1^ primers (SANGON BIOTECH, Shanghai, China) (Table S1), and 1 μL of each template, resulting in a total volume of 20 μL. The PCR program was: 95 °C for 3 min; 40 cycles of 94 °C for 15 s, 60 °C for 20 s, and 72 °C for 15 s. All the samples were analyzed with three biological replicates, each comprising three technical replicates. Relative gene expression levels were calculated in accordance with the 2^−ΔΔCt^ method^30^.

### Genetic transformation

Genetic transformations were performed by agrobacterium infection methods in accordance with published methods for *Arabidopsis*^31^ and tomato (‘Ailsa Craig’)^32^ plants. The transgenic *Arabidopsis* and tomato lines were grown on MS plates supplemented with 50 mg L^−1^ and 100 mg L^−1^ kanamycin, respectively.

### Salt- and mannitol-tolerance assays

The ‘Fuji’ plants at 30 d after propagation were used for the 150 mmol L^−1^ salt and 300 mmol L^−1^ mannitol treatment. We collected leaf samples before and at 1, 3, and 6 h after each of the two treatments. The samples were immediately frozen in liquid nitrogen and stored at − 80 °C.

The transgenic *Arabidopsis* seeds were grown in 1/2MS medium containing 75 and 100 mmol L^−1^ salt, and 100 and 200 mmol L^−1^ mannitol. The transgenic tomato seeds were grown in MS medium containing 100 mmol L^−1^ salt. On the 8th day after germination, we calculated the root length and the fresh weight of the seedlings.

### Statistical analyses

Statistical analyses were performed using SPSS software. Data are reported as means ± SDs. Asterisks (*) indicate significant differences between treatments as assessed using Student’s t-test at *P* < 0.05 (*) and *P* < 0.01 (**). Different lowercase letters above the bars indicate significant differences (*P* < 0.05, Tukey’s test).

### Ethics approval and consent to participate

Prior to conducting the research, the permission from Hebei Normal University of Science and Technology and Horticulture College of Northwest A & F University to collect and analyse the Fuji, *Arabidopsis*, and Tomato sample documented in this work was obtained. All the experimental materials in this study do not violate the IUCN Policy Statement on Research Involving Species at Risk of Extinction and Convention on the Trade in Endangered Species of Wild Fauna and Flora, and have been approved by the government.

## Results

### Expression analysis of ***MdNup62***

**Figure 1:**
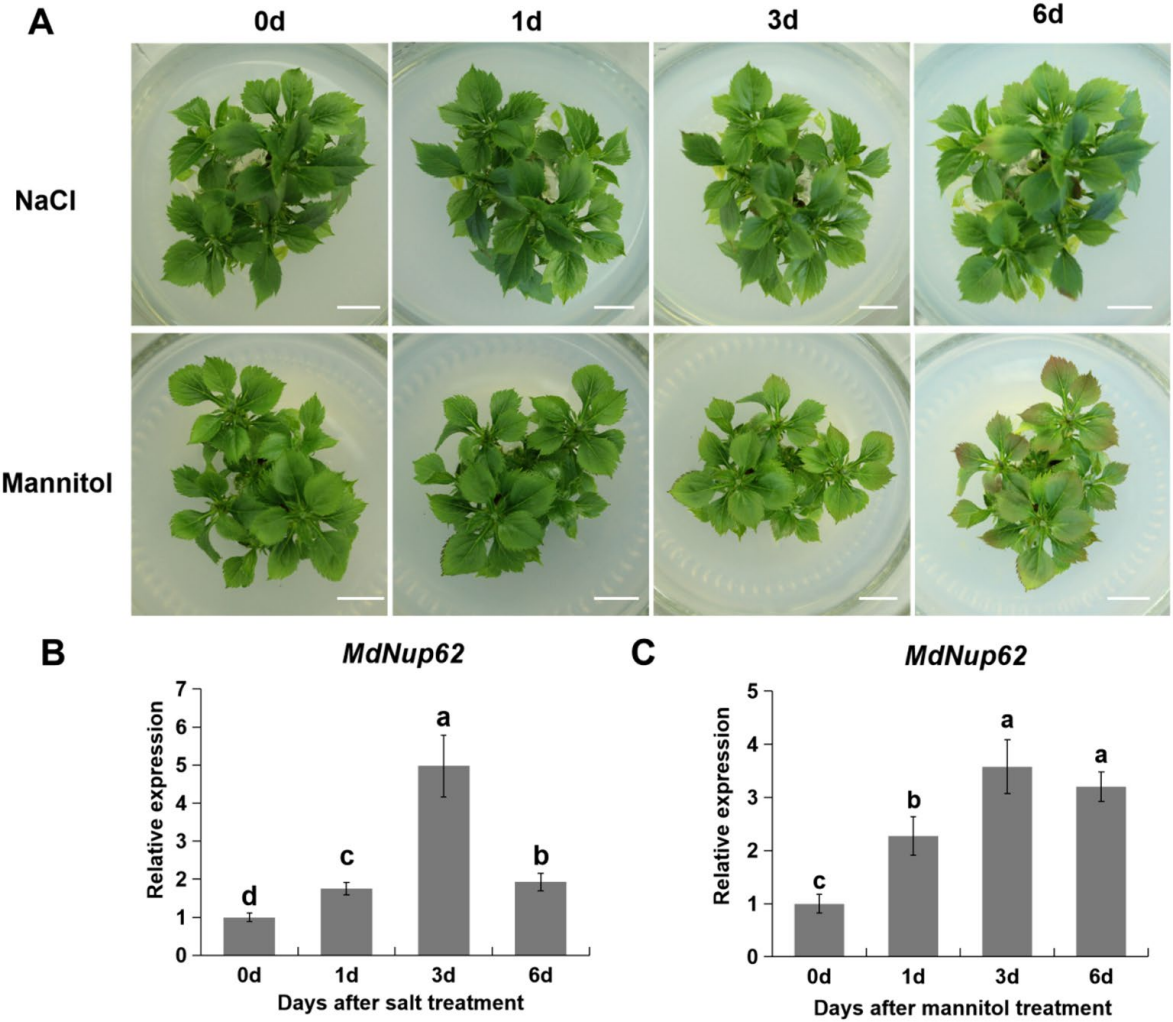
Expresion analysis of *MdNup62* under salt and mannitol treatment. (**A**) The phenotype of ‘Nagafu No. 2’ tissue-cultured seedlings at 0, 1, 3, and 6 h under salt(upper panels) and mannitol(lower panels) treatment conditions. Bar = 1 cm. (**B**) and (**C**) Analyses of *MdNup62* expression levels in ‘Nagafu No. 2’ tissue-cultured seedling leaves at 0, 1, 3, and 6 h under (**B**) salt and (**C**) mannitol treatment conditions. Each sample was analysed with three biological replicates, each comprising three technical replicates. Means followed by different lowercase letters are significantly different at the 0.05 level. The same below.

We exposed apple tissue-cultured seedlings to salt and mannitol treatments. *MdNup62* expression level was determined at different times during the salt and mannitol treatments (Fig. 1). *MdNup62* was significantly induced by salt, and its expression level was highest at 3 d after salt treatment (Fig. 1B). *MdNup62* was also significantly induced by mannitol; its expression level was highest at 3 d and 6 d after mannitol treatment (Fig. 1C).

### Overexpression of* MdNup62* reduces salt-stress tolerance

**Figure 2:**
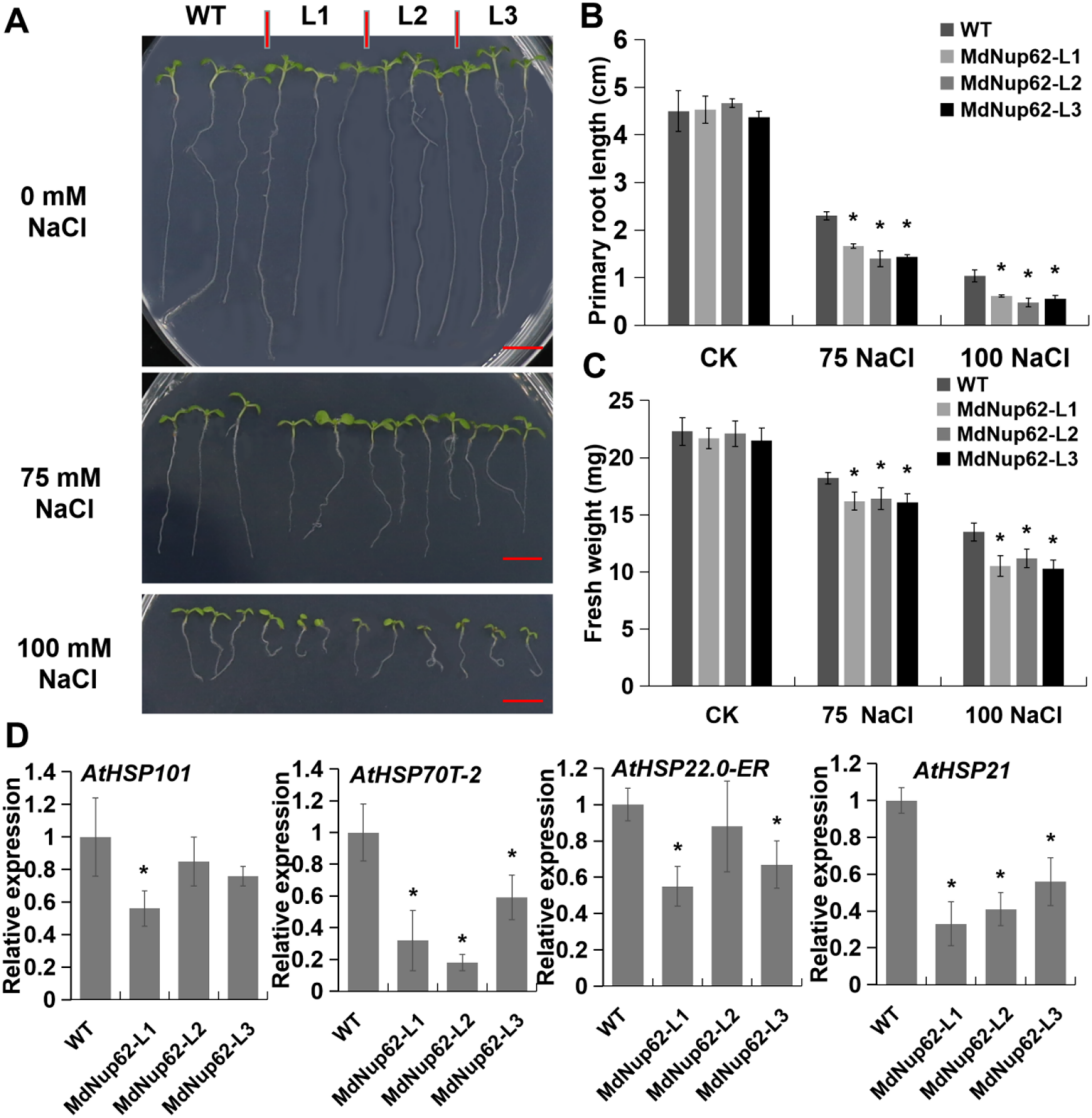
*MdNup62* reduced salt-stress tolerance in *Arabidopsis*. (**A**) Phenotype of the *MdNup62*-overexpression *Arabidopsis* lines for salt-stress tolerance. Bar = 1 cm. (**B**) and (**C**) Statistical analysis of (**B**) primary root length and (**C**) fresh weight of WT and *MdNup62*-overexpression *Arabidopsis* lines after the salt treatment. Asterisks denote significant differences as determined by a t-test (*P < 0.05). (**D**) qRT-PCR analysis of four *AtHSPs* expression in *Arabidopsis* samples after 100 mM NaCl treatment. Asterisks denote significant differences as determined by a t-test (*P < 0.05).The same below.

To confirm the role of *MdNup62*'s in salt-stress tolerance, we performed an Agrobacterium-mediated genetic transformation of *MdNup62* into *Arabidopsis*. We found that the root length of *MdNup62*-OE lines were significantly shorter and their fresh weight were lower than that of WT on MS culture medium with both 75 and 100 mM salt treatment, whereas there was no significant difference in root length and fresh weight without salt treatment (Fig. 2A–C). The presence of the transgene in *MdNup62*-OE lines was confirmed using qRT-PCR (Figure S1A). Because our previous research found that MdNup62 interacts with some MdHSFs^29^, and *HSPs* are located downstream of *HSFs*. So we performed a qRT-PCR analysis of four *AtHSPs* (*AtHSP101*, *AtHSP70T-2, AtHSP22.0-ER*, and *AtHSP21*) (Fig. 2D). Their expression levels in transgenic *Arabidopsis* were reduced under salt-stress conditions.

**Figure 3:**
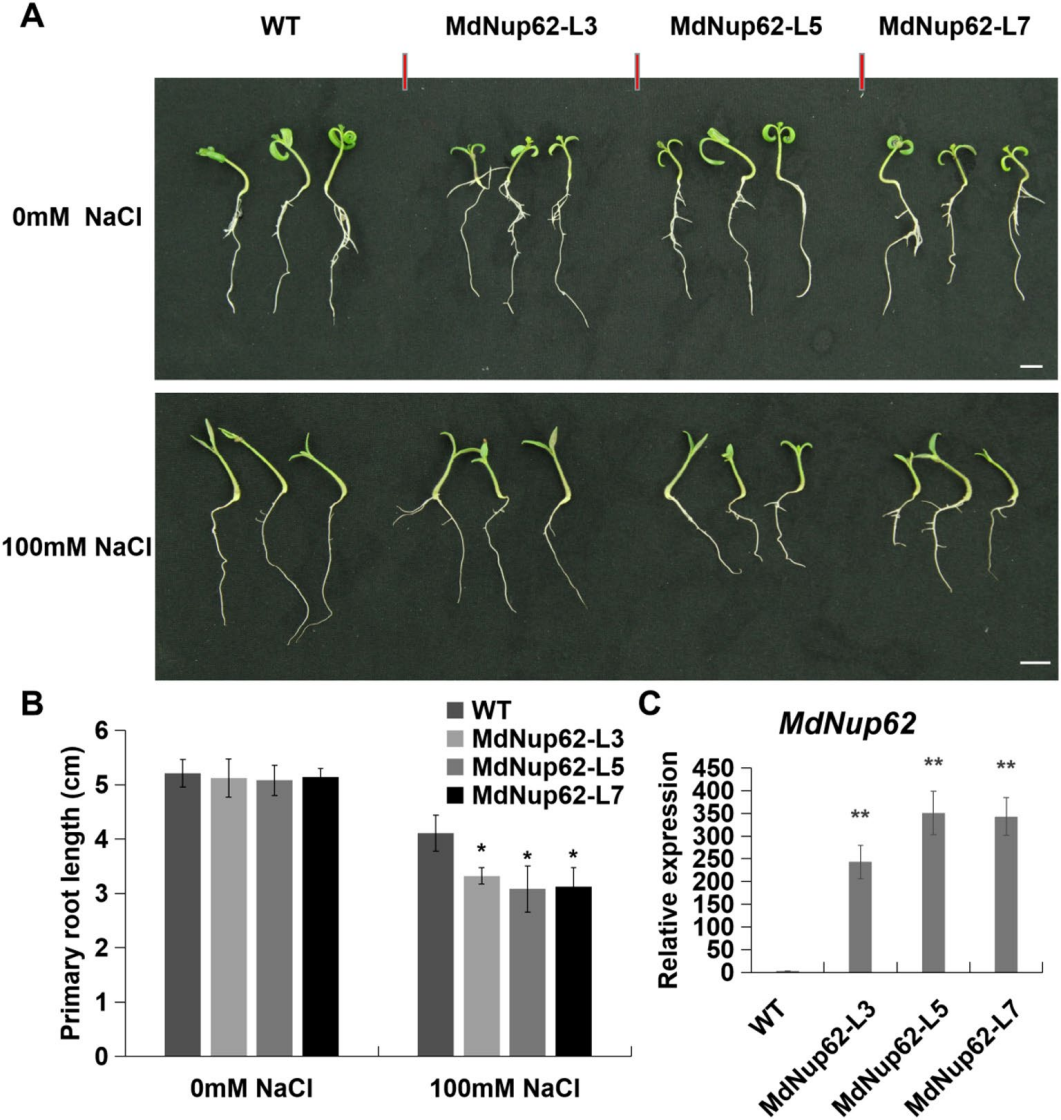
*MdNup62* reduced salt-stress tolerance in tomato. (**A**) Phenotype of the *MdNup62*-overexpression tomato lines for salt-stress tolerance. Bar = 1 cm. (**B**) Statistical analysis of primary root length of WT and *MdNup62*-overexpression tomato lines after the salt treatment. (**C**) qRT-PCR analysis of *MdNup62* expression in tomato samples.

Moreover, salt-stress tolerance assays were carried out in transgenic tomato plants (Fig. 3). As in transgenic *Arabidopsis*, the root length of transgenic tomato was significantly reduced compared with that of WT (Fig. 3A,B). The presence of the transgene in *MdNup62*-OE lines was confirmed by qRT-PCR (Fig. 3C).

### *MdNup62* overexpression reduces osmotic-stress tolerance

**Figure 4:**
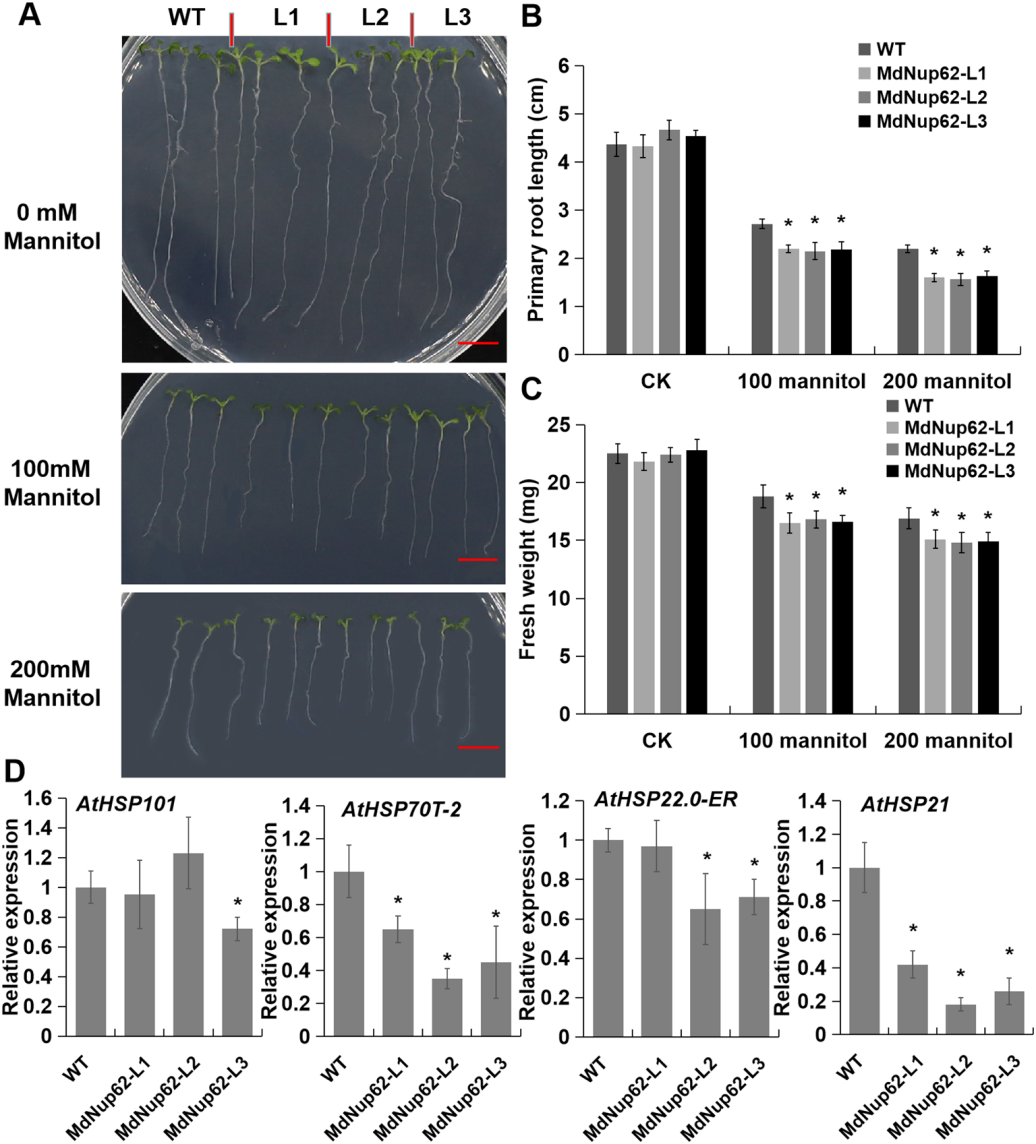
*MdNup62* reduced osmotic-stress tolerance in *Arabidopsis*. (**A**) Phenotype of the *MdNup62*-overexpression *Arabidopsis* lines for osmotic-stress tolerance. Bar = 1 cm. (**B**) and (**C**) Statistical analysis of (**B**) primary root length and (**C**) fresh weight of WT and *MdNup62*-overexpression *Arabidopsis* lines after the mannitol treatment. (**D**) qRT-PCR analysis of four *AtHSPs* expression in *Arabidopsis* samples after 200 mM mannitol treatment.

We studied the phenotype of *MdNup62*-OE lines in *Arabidopsis* under osmotic (mannitol) treatment and obtained same results to those of salt-stress treatment. The root length of *MdNup62*-OE lines were significantly shorter and their fresh weight were lower than that of WT on MS culture medium with both 100 and 200 mM mannitol treatment, whereas there was no significant difference in root length and fresh weight without mannitol treatment (Fig. 4A–C). And the expression levels of the four *AtHSPs* (*AtHSP101*, *AtHSP70T-2, AtHSP22.0-ER*, and *AtHSP21*) in transgenic *Arabidopsis* were reduced under osmotic-stress conditions (Fig. 4D).

### Sequence and expression analysis of *MdHSFA1d*

**Figure 5:**
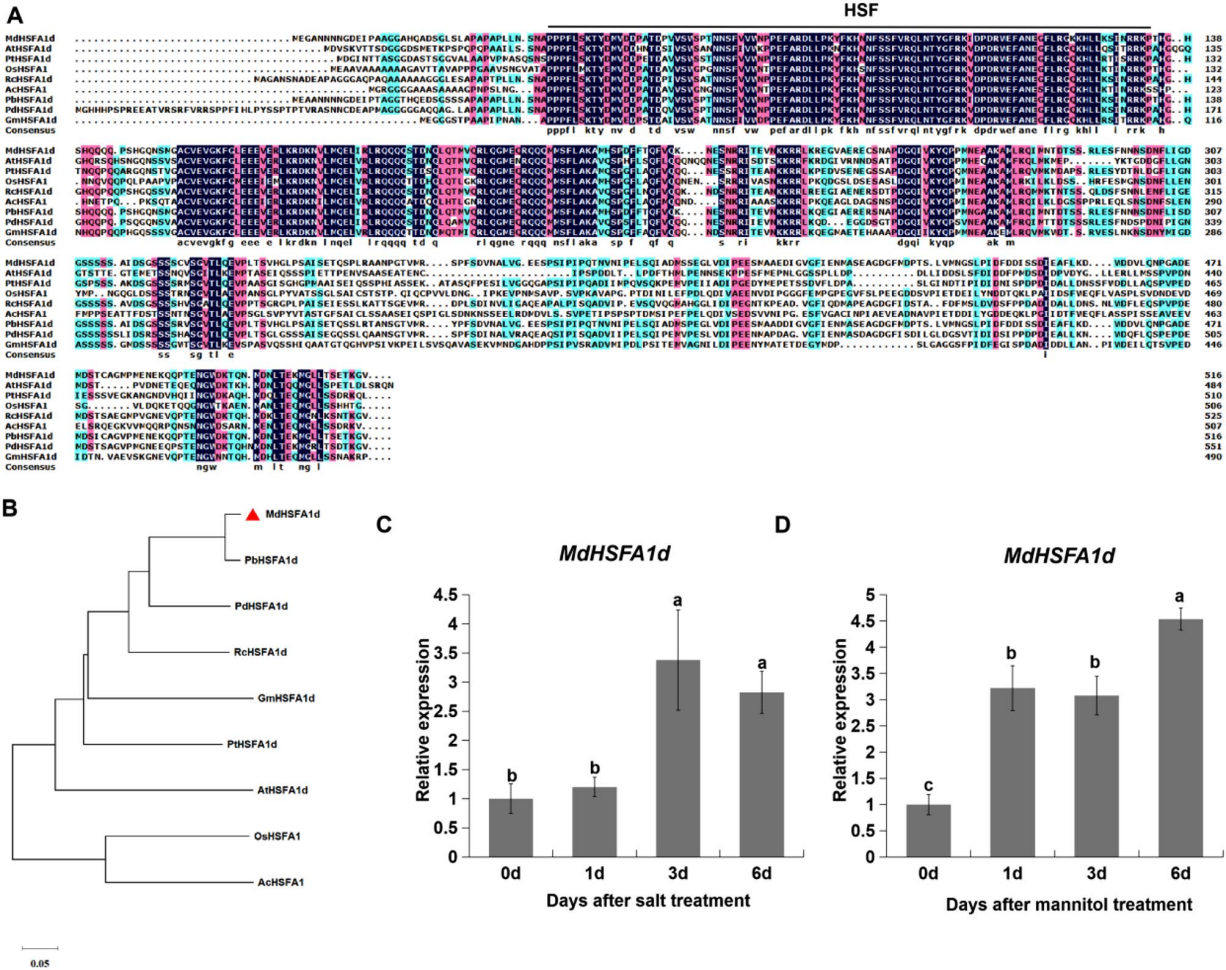
Bioinformatics and expresion analysis of *MdHSFA1d*. (**A**) The conservative domain of HSFA1d in 9 plant species (*Arabidopsis*, *Malus domestica*, *Populus trichocarpa*, *Oryza sativa*, *Rosa chinensis*, *Pyrus bretschneideri*, *Ananas comosus*, *Prunus dulcis*, and *Glycine max*). (**B**) The phylogenetic tree of *HSFA1d* from 9 species. (**C**) and (**D**) Analyses of *MdHSFA1d* expression levels in ‘Nagafu No. 2’ tissue-cultured seedling leaves at 0, 1, 3, and 6 d under (**C**) salt and (**D**) mannitol treatment conditions.

Because our previous research found that MdNup62 interacts with MdHSFs^29^, we chose one of the interacting proteins, MdHSFA1d, to investigate its relationship with the function of MdNup62. And we performed the sequence analysis of MdHSFA1d first. Multiple sequence alignment of HSFA1d homologs of nine plant species (*Arabidopsis*, *Malus domestica*, *Populus trichocarpa*, *Oryza sativa*, *Rosa chinensis*, *Pyrus bretschneideri*, *Ananas comosus*, *Prunus dulcis*, and *Glycine max*) revealed a universally conserved HSF domain (Fig. 5A). Phylogenetic analysis indicated that MdHSFA1d was closely related to genes in *Pyrus bretschneideri*, *Prunus dulcis*, and *Rosa chinensis* in the family Rosaceae, but is more distantly related to genes in monocotyledons (*Oryza sativa* and *Ananas comosus*) (Fig. 5B). *MdHSFA1d* expression was determined at different times under the salt and mannitol treatments (Fig. 5C,D). *MdHSFA1d* was significantly induced by salt, and its expression level was highest at 3 d and 6 d after salt treatment. *MdHSFA1d* was also significantly induced by mannitol, with expression levels highest at 6 d after mannitol treatment.

### *MdHSFA1d* overexpression promotes salt-stress tolerance

**Figure 6:**
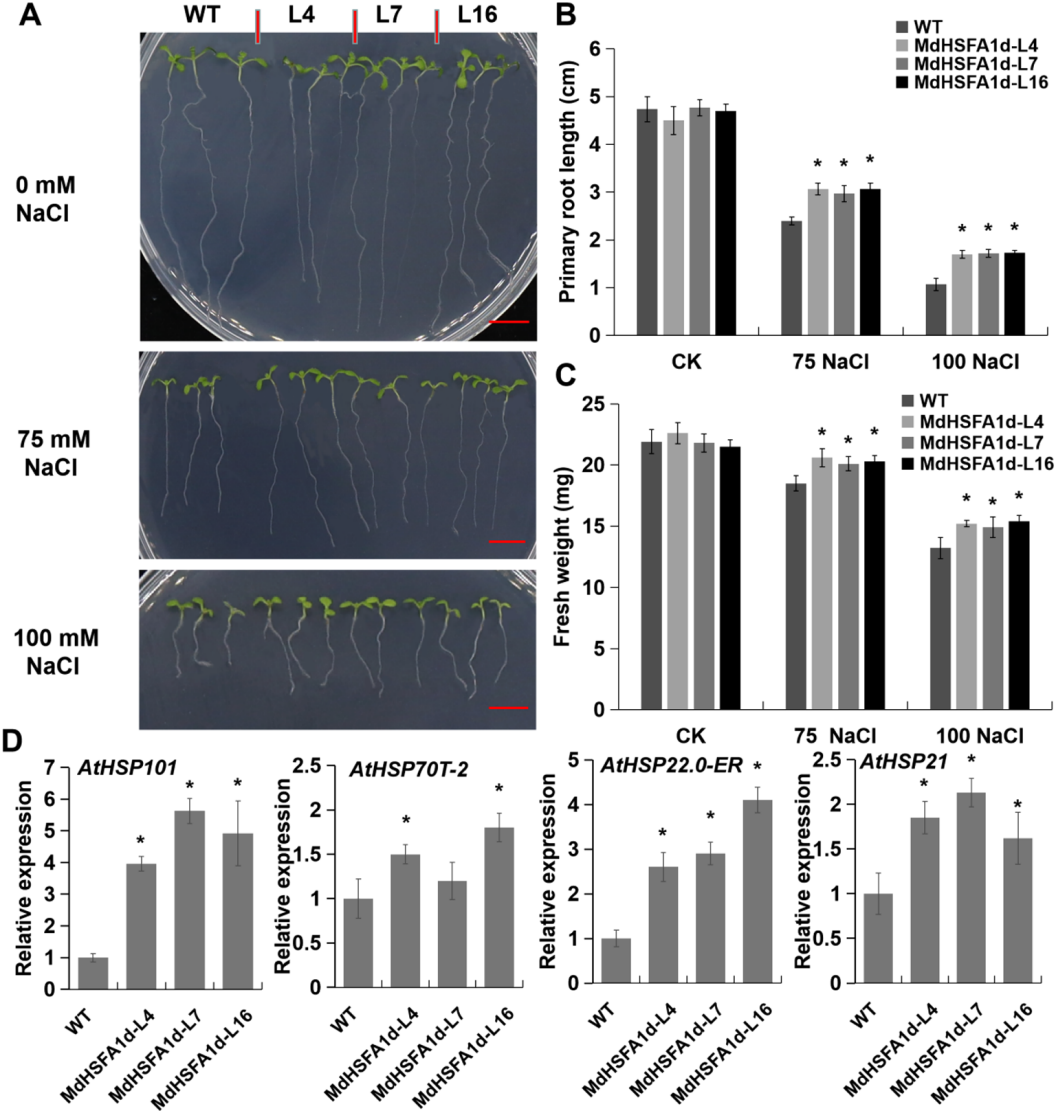
*MdHSFA1d* promotes salt-stress tolerance in *Arabidopsis*. (**A**) Phenotype of the *MdHSFA1d*-overexpression *Arabidopsis* lines for salt-stress tolerance. Bar = 1 cm. (**B**) and (**C**) Statistical analysis of (**B**) primary root length and (**C**) fresh weight of WT and *MdHSFA1d*-overexpression *Arabidopsis* lines after the salt treatment. (**D**) qRT-PCR analysis of four *AtHSPs* expression in *Arabidopsis* samples after 100 mM NaCl treatment.

To verify the salt-stress tolerance phenotype of *MdHSFA1d*, we performed Agrobacterium-mediated genetic transformations of *MdHSFA1d* into *Arabidopsis*. The root length of *MdHSFA1d*-OE lines were significantly longer and their fresh weight were higher than that of WT on MS culture medium with both 75 and 100 mM salt treatment, whereas there was no significant difference in root length and fresh weight without salt treatment (Fig. 6A–C). The presence of the transgene in *MdHSFA1d*-OE lines was confirmed using qRT-PCR (Figure S1B). And a qRT-PCR analysis of four *AtHSPs* (*AtHSP101*, *AtHSP70T-2, AtHSP22.0-ER*, and *AtHSP21*) were also performed (Fig. 6D). Their expression levels in transgenic *Arabidopsis* were increased under salt-stress conditions.

### *MdHSFA1d* overexpression promotes osmotic-stress tolerance

**Figure 7:**
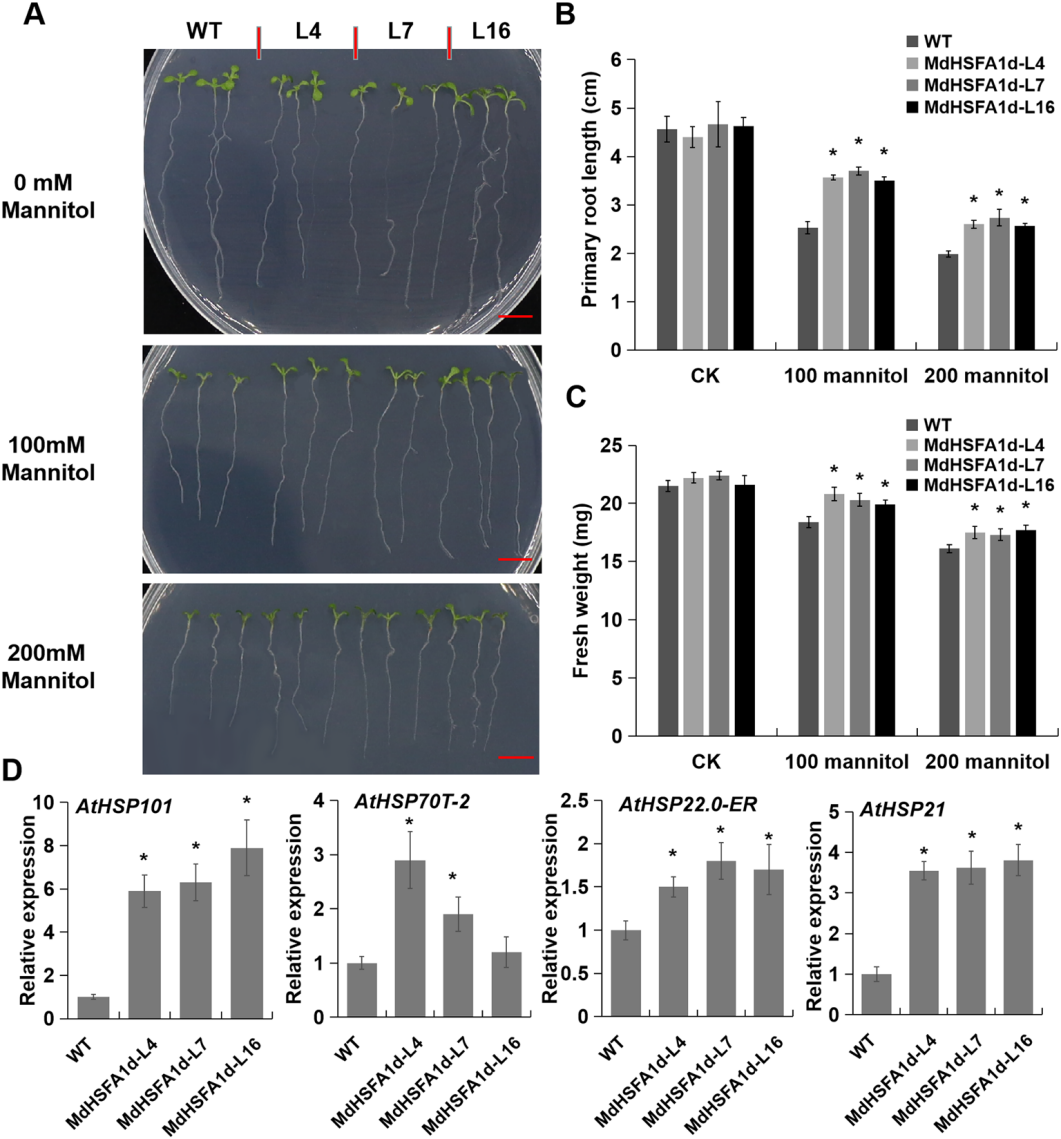
*MdHSFA1d* promotes osmotic-stress tolerance in *Arabidopsis*. (**A**) Phenotype of the *MdHSFA1d*-overexpression *Arabidopsis* lines for osmotic-stress tolerance. Bar = 1 cm. (**B**) and (**C**) Statistical analysis of (**B**) primary root length and (**C**) fresh weight of WT and *MdHSFA1d*-overexpression *Arabidopsis* lines after the mannitol treatment. (**D**) qRT-PCR analysis of four *AtHSPs* expression in *Arabidopsis* samples after 200 mM mannitol treatment.

To study the osmotic-stress tolerance phenotypes of MdHSFA1d, we exposed *MdHSFA1d*-OE transgenic plants to osmotic-stress (mannitol). The root length of *MdHSFA1d*-OE lines were significantly longer and their fresh weight were higher than that of WT on MS culture medium with both 100 and 200 mM mannitol treatment, whereas there was no significant difference in root length and fresh weight without mannitol treatment (Fig. 7A–C). And the expression levels of four *AtHSPs* (*AtHSP101*, *AtHSP70T-2, AtHSP22.0-ER*, and *AtHSP21*) in transgenic *Arabidopsis* were increased under osmotic-stress conditions (Fig. 7D).

## Discussion

Although the key factors of abiotic stress signaling in plants have been identified, the abiotic stress response is complex and several other factors have not been discovered yet. As channels of material communication between the cytoplasm and nucleus, nucleoporins are involved in the regulation of abiotic stress pathways in plants. Nucleoporin *CPR5*, *HOS1*, and *Nup160* are involved in the temperature stress pathway^14, 16, 33^. The expression of ABA and salt-induced stress response genes, such as *RESPONSIVE TO DESICCATION 29A (RD29A)*, *COLD-REGULATED 47 (COR47)*, *COR15A*, was significantly decreased in *nup85*, *nup160*, and *hos1* mutant lines. MEDIATOR SUBUNIT 18 (MED18) and Nup85 exhibit direct interaction with each other, and both *MED18* and *Nup85* mutants show increased sensitivity to ABA and salt stress with attenuated expression of stress response genes^15^. In our previous study, we found that overexpression of apple *MdNup62* significantly reduced heat tolerance in *Arabidopsis*^29^. In the present study, *MdNup62* was significantly induced by salt and mannitol (osmotic simulation) treatments (Fig. 1). Moreover, the *MdNup62*-OE *Arabidopsis* lines exhibited significantly reduced salt and osmotic tolerance (Figs. 2, 4). Many HSP genes have been reported to be associated with salt- and osmotic-stress. For example, HSP70, HSP40, HSP17.0, and HSP23.7 were positive regulating salt-stress tolerance^34–36^, while HSP70,and HSP90 were related to osmotic-stress tolerance^37, 38^. And in this study, we found that four *AtHSPs* (*AtHSP101*, *AtHSP70T-2, AtHSP22.0-ER*, and *AtHSP21*) in *MdNup62*-OE *Arabidopsis* lines were reduced under salt- and osmotic-stress conditions (Figs. 2, 4). So, we speculate that *MdNup62*-OE *Arabidopsis* lines negatively regulate salt- and osmotic-stress tolerance by reducing the expression level of HSP genes. These results indicate that *MdNup62* is a factor in salt and osmotic stress responses in apple.

HSFs are involved in the regulation of several abiotic stress pathways. For example, *Arabidopsis AtHSFA6a* positively affects the regulation of drought and salt tolerance, whereas rice *OsHSFB2b* and maize *ZmHSF08* exhibit negative regulatory effects^23, 24, 39^. Apple *MdHSFA8* enhanced plant viability under drought conditions by promoting the accumulation of flavonoids and scavenging reactive oxygen species^22^. In our previous study, we found that both *MdHSFA1d*-OE and *MdHSFA9b*-OE *Arabidopsis* exhibited significantly increased heat tolerance^29^; similar results were obtained in this study. *MdHSFA1d* was significantly induced by salt and mannitol (osmotic simulation) treatments (Fig. 5). When treated with salt and mannitol, the *MdHSFA1d*-OE *Arabidopsis* exhibited better growth (Figs. 6, 7). The HSP genes are located downstream of the HSF transcription factors and are directly or indirectly regulated by them^40^. Unlike *MdNup62*-OE *Arabidopsis* lines, these four *AtHSPs* (*AtHSP101*, *AtHSP70T-2, AtHSP22.0-ER*, and *AtHSP21*) in *MdHSFA1d*-OE *Arabidopsis* lines were increased under salt- and osmotic-stress conditions (Figs. 6, 7). This may be an important reason for the phenotypic differences between the two transgenic lines. These results indicate that *MdHSFA1d* is also a factor in salt and osmotic stress responses in apple.

Apple MdNup62 and MdHSFA1d proteins interact directly with each other and present opposite phenotypes under high temperature stress^29^; their overexpression in *Arabidopsis* also presents opposite phenotypes under salt and osmotic stress conditions. Therefore, we hypothesized that MdNup62, as a nucleoporin, may hinder HSF transport and negatively affect the regulation of salt and osmotic stress responses. However, previous studies found that both *Nup62* deletion and overexpression strains of *Arabidopsis* exhibit increased sensitivity to auxin, indicating that overexpression does not result in functional gain, but rather functional loss^41^. With osmotic and salt treatments, apple *MdHSFA1d* was significantly induced and transported into the nucleus through NPC channels to promote downstream HSPs expression in WT, thereby enhancing osmotic and salt tolerance. In *MdNup62-*OE lines, the structure of the apple NPC changed, blocking the transport of MdHSFA1d into the nucleus and causing plant injury. Based on these results, we inferred that apple MdNup62 is involved in the regulation of salt and osmotic stress responses by controlling the transport of MdHSFA1d between the nucleus and cytoplasm.

### Supplementary Information

Supplementary Information.

## Data Availability

All data generated or analysed during this study are included in this published article [and its supplementary information files].
