# Supplementary material for: MdNup62 involved in salt and osmotic stress tolerance in apple
Source: Sci Rep. 2023 Nov 18;13:20198. doi: 10.1038/s41598-023-47024-9 (PMC10657396; doi:10.1038/s41598-023-47024-9)
Supplement: Supplementary file 1 — Supplementary Information. [file 41598_2023_47024_MOESM1_ESM.docx]

**MdNup62 involved in salt and osmotic stress tolerance in apple**

Ruxuan Guo^1^†, Xiaoshuang Zhang^1^†, Mingyuan Li^1^, Huiwen Zhang^1^, Junkai Wu^1^, Libin Zhang^1^, Xiao Xiao^1^, Mingyu Han^2^, Na An^2^, Libo Xing^2^, Chenguang Zhang^1^*

^1^Hebei Key Laboratory of Horticultural Germplasm Excavation and Innovative Utilization, College of Horticulture Technology, Hebei Normal University Of Science & Technology, 066600, Changli, Hebei, P. R. China

^2^College of Horticulture, Northwest A&F University, 712100 Yangling, Shaanxi, P. R. China

**†Equal contributors**

***Corresponding author:**

Chenguang Zhang

E-mail: Zchenguang0105@163.com;

Tel.: +8616630500117;

Address: Huiwen street, Changli 066600, Hebei, P. R. China

**Table S1**

| **Gene name** | **Gene ID** | **Primer sequences** |
| --- | --- | --- |
| *AtActin*-F | AT2G37620 | GCGATTCCGTTGTCCTGAGGTTC |
| *AtActin*-R | AT2G37620 | TTCCACCACTGAGCACAATGTTACC |
| AtHSP101-F | AT1G74310 | AAGATGGTTGTGCGTGAG |
| AtHSP101-R | AT1G74310 | CTGACTTCTTGCCTGTTGA |
| AtHSP22.0-ER-F | AT4G10250 | ACCAGAGAATTGAGACTAACC |
| AtHSP22.0-ER-R | AT4G10250 | GCTCCTATGAAGAAGATGCT |
| AtHSP70T-2-F | AT2G32120 | ACAGCAGCAGATGACTAC |
| AtHSP70T-2-R | AT2G32120 | TAGCAGTAACCGCAACAT |
| AtHSP21-F | AT4G27670 | GGCTTCTACACTCTCATTTGC |
| AtHSP21-R | AT4G27670 | GGTCTTGAGCCCTGATCC |
| AtRD29A-F | AT5G52310 | GCCGACGGGATTTGACG |
| AtRD29A-R | AT5G52310 | GCCGGAAATTTATCCTCTTCTGA |
| *SlActin*-F | Solyc11g005330 | GTCCTCTTCCAGCCATCCATGA |
| *SlActin*-R | Solyc11g005330 | ACCACTGAGCACAATGTTACCG |
| *MdActin*-F | MD04G1127400 | CAACTCATCCGAACCTCAAACC |
| *MdActin*-R | MD04G1127400 | CGCTGTCCGCCATCTTCTACT |
| *MdHSFA1d*-F | MD16G1271200 | ACTTCTTGATTGGTGATGGTTC |
| *MdHSFA1d*-R | MD16G1271200 | CCGCAGAGGAGATTGTGTT |
| *MdNUP62*-F | MD07G1110700 | GGCTTCCTCCGCTTCACAATCC |
| *MdNUP62*-R | MD07G1110700 | CGGTGCTAGGAGCCTGAGAAGT |


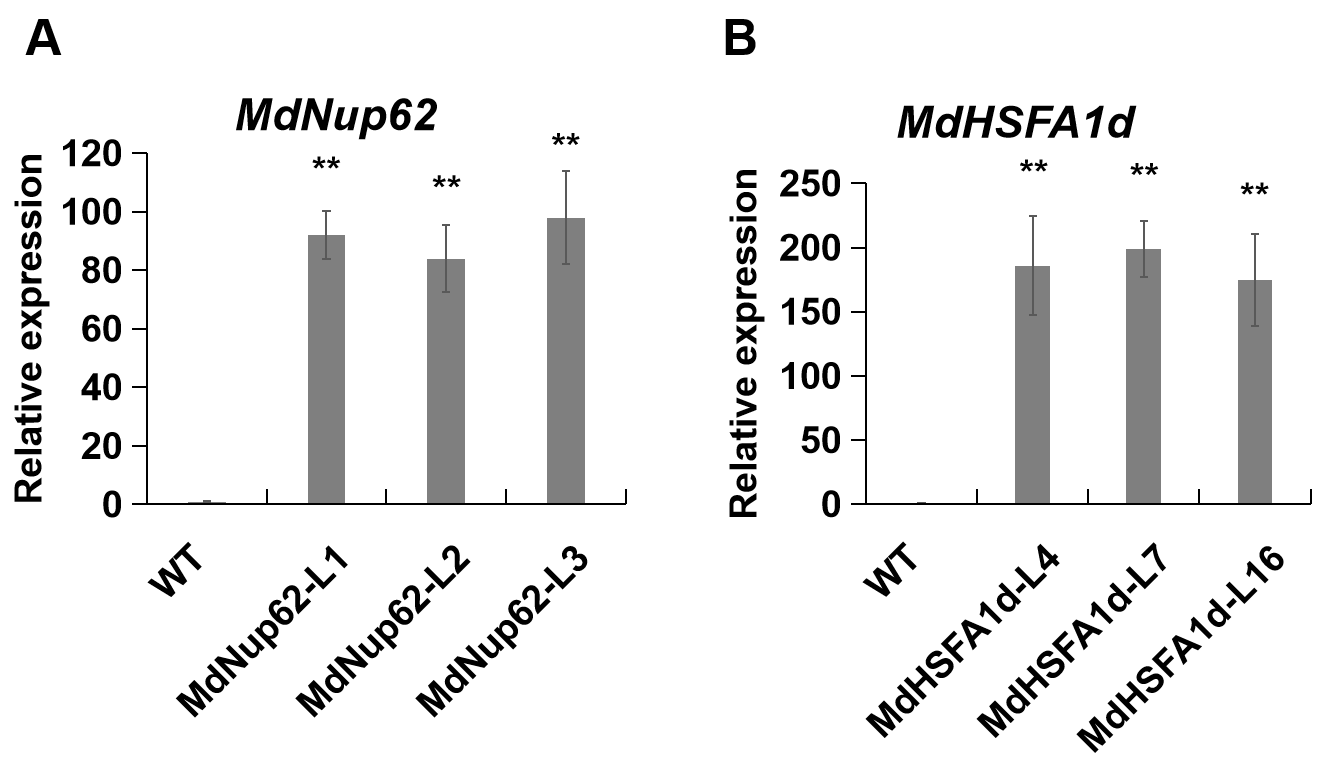


**Figure S1**
